# Narrowing the spectrum: the new frontier of precision antimicrobials

**DOI:** 10.1186/s13073-017-0504-3

**Published:** 2017-12-14

**Authors:** Alexandra E. Paharik, Henry L. Schreiber, Caitlin N. Spaulding, Karen W. Dodson, Scott J. Hultgren

**Affiliations:** 1 Department of Molecular Microbiology, Washington University School of Medicine, St. Louis, MO 63110 USA; 2 Department of Immunology and Infectious Diseases, Harvard T.H. Chan School of Public Health, Boston, MA 02115 USA; 3 Center for Women’s Infectious Disease Research, Washington University School of Medicine, St. Louis, MO 63110 USA

## Abstract

Antibiotics have become the standard of care for bacterial infections. However, rising rates of antibiotic-resistant infections are outpacing the development of new antimicrobials. Broad-spectrum antibiotics also harm beneficial microbial communities inhabiting humans. To combat antibiotic resistance and protect these communities, new precision antimicrobials must be engineered to target specific pathogens.

## The microbiota, human health, and effects of antibiotics

Alexander Fleming’s serendipitous discovery of the antibiotic penicillin in 1929, and the subsequent discovery of streptomycin in 1943, ushered in the golden age of antibiotic discovery (1950s–1970s), in which approximately half of the antibacterial drugs commonly used today were discovered. Since this time period, antibiotics have become the standard of care for bacterial infections. Antibiotics greatly reduce the morbidity and mortality of infectious disease, and increase the quality and length of life for billions of people. However, bacterial resistance to antimicrobial drugs followed shortly after their development, and is currently a global health crisis. A lack of stewardship in the use of broad-spectrum antimicrobials, both in healthcare and farming settings, has led to a precipitous increase in the occurrence of antibiotic-resistant microorganisms [1]. Broad-spectrum antimicrobials expose the resident human microbiota (the collection of microorganisms living in or on the human body) to selective pressure, and failure to complete a course of antibiotics leads to incomplete eradication of infectious microorganisms and the development of resistance in surviving pathogens. Commensal microbes of livestock are also affected by the use of antibiotics, which are used as growth promoters and as treatment for infections. Foodborne transfer then allows resistant microorganisms to colonize humans. Furthermore, bottlenecks in the discovery and clinical testing of novel antibiotics have led to a dearth of new antimicrobial drugs in the pipeline. Thus, infections caused by drug-resistant bacteria are currently outpacing the development of new antimicrobial drugs, and are threatening to again make common infections a life-or-death problem.

An increasing number of studies reveal that the broad-spectrum nature of antibiotics and their overuse have long-lasting detrimental effects on the healthy human microbiota, which has important functions in metabolism, resistance to pathogens, and immune system development [2, 3]. For instance, the healthy gut microbiota confers colonization resistance to invading pathogens and plays vital roles in nutrient acquisition and modulation of the immune system [2]. Disruption of the community structure, and thus the function, of the microbiota is known as dysbiosis, and has been linked to multiple immunological and metabolic diseases [2, 3].

In young children, exposure to antibiotics could be particularly damaging, as maturation of the gut microbiota community is crucial for healthy childhood development, impacting the growth of muscle, adipose, and bone tissue, and the development of a healthy immune system [3]. In adults, prolonged antibiotic use can also result in decreased gut microbial diversity and increased susceptibility to the gastrointestinal pathogen *Clostridium difficile.* When *C. difficile* infections are treated with further antibiotics, recurrent infection rates can be as high as 65% [4]. Thus, although broad-spectrum antibiotics play a critical role in saving lives and curing infection, their use can result in long-term, detrimental effects and damage to the human microbiota.

## Precision antimicrobials: novel developments and challenges

An alternative to broad-spectrum antibiotics is precision antimicrobials. Precision antimicrobials function either by i) specifically inhibiting a critical node in pathogenesis to disrupt maintenance and/or persistence of the pathogen in the host, or by ii) specifically killing the pathogenic organism with minimal off-target effects. Such strategies are less likely to induce resistance than broad-spectrum antimicrobials, since targeting key factors that are required for virulence in specific bacteria limits the ways that bacteria can develop resistance while maintaining virulence function. Furthermore, resident microorganisms are less likely to develop resistance to pathogen-targeted therapies as they do not employ the same biochemical pathways.

**Fig. 1:**
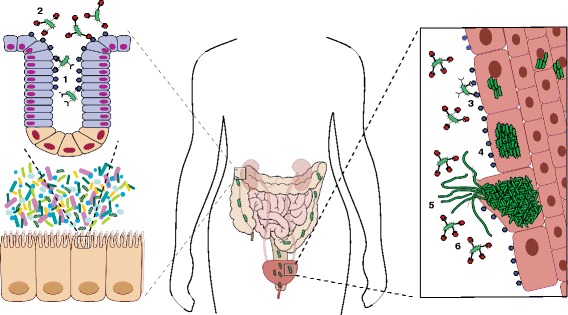
Antibiotic-sparing mannoside simultaneously treats an active bladder infection and targets the gastrointestinal reservoir of uropathogenic *E. coli*. Uropathogenic *E. coli* (UPEC) binds mannosylated proteins (*blue*) found on the epithelia of the gut and bladder. *1* In the gut, UPEC bind within the colonic crypts via interactions between the FimH adhesin on type I pili and mannose. *2* Mannosides (*red*) bind FimH with greater affinity than mannose, removing colonizing UPEC from the gastrointestinal tract. *3–5* The infection cycle of a urinary tract infection involves multiple stages, including initial attachment (*3*), intracellular proliferation (*4*), filamentation and efflux (*5*), and re-entry. Attachment and re-entry require FimH to bind mannose (*blue*) on the bladder epithelium. *6* Mannosides (*red*) bind FimH and prevent binding to bladder cells, promoting elimination of UPEC from the tissue. UPEC, Uropathogenic *Escherichia coli*

Biomedical research is devoting great efforts to the development of next-generation precision antimicrobials for the world’s most prevalent pathogens, particularly for those diseases with highly resistant pathogens. For instance, the drug resistance index for urinary tract infections (UTIs) shows that the number of infections that face treatment difficulties has increased since the mid-2000s due to the rapid spread of resistance among Gram-negative microorganisms, which includes *Escherichia coli*, the primary cause of UTIs [5]. A recent study by Spaulding et al. [6] exemplified how the use of precision antimicrobials could help to thwart this problem. Uropathogenic *E. coli* (UPEC), which cause the majority of UTIs, reside asymptomatically in an intestinal reservoir. UPEC are shed in the feces, can colonize the periurethral area, and then ascend the urethra to cause a UTI. Type 1 pili tipped with the FimH adhesin facilitate UPEC colonization of both the gut and the bladder by binding mannosylated proteins that decorate the gut and bladder epithelia (Fig. 1) [6]. Substituted analogs of mannose, called mannosides, have been developed to specifically block the ability of UPEC to colonize the host by binding to *E. coli* FimH [7]. The optimal analogs are biphenyl mannosides, which bind FimH with orders of magnitude higher affinity (~ 1,000,000×) than the natural receptor [7]. Spaulding et al. [6] showed that biphenyl mannosides were not only effective in treating an active bladder infection, but also were able to simultaneously reduce colonization of UPEC in the gastrointestinal tract of mice, while leaving the structure of the microbial community undisturbed (Fig. 1) [6]. Thus, this antibiotic-sparing therapy could prevent recurrent UTIs by both reducing UPEC persistence within the host intestinal reservoir and by preventing colonization of the bladder. Furthermore, mutations in *fimH* that confer resistance to mannoside binding would likely also disrupt its crucial interactions with mannosylated host proteins. Mannosides are therefore a promising therapeutic candidate with low selection pressure for resistance.

Avidocin-CDs are also an example of a precision antibiotic that does not disrupt the gut microbiota. Kirk et al. [8] demonstrated that the Avidocin-CD class of bactericides specifically kills *C. difficile* by targeting SlpA, the primary component of the *C. difficile* S-layer. Various forms of Avidocin-CDs can be designed that kill particular isolates of *C. difficile* based on the S-layer type [8]. Although S-layer-null mutants that are resistant to this bactericide have been identified in vitro, they are avirulent, which demonstrates the importance of the S-layer in *C. difficile* sporulation and toxin production [8]. By showing that resistance to Avidocin-CDs forces *C. difficile* to lose virulence, this work provides an elegant example of the advantages to designing bactericides that target virulence factors.

Successful precision therapeutics may also target a variety of other virulence pathways, such as toxin production. Small molecules virstatin and toxtazin B are antivirulence inhibitors of *Vibrio cholerae* toxin expression, and both are efficacious in animal models of *V. cholerae* infection [9]. The antivirulence drug bezlotoxumab, a monoclonal antibody against the *C. difficile* toxin TcdB, was FDA-approved in 2016 to treat *C. difficile* patients at high risk of recurrent infection [10]. The FDA has also approved the use of therapeutics that neutralize *Clostridium botulinum* neurotoxins (BoNTs) and *Bacillus anthracis* protective antigen, a component of both the lethal toxin and the edema toxin [10].

There are a number of important considerations inherent in the successful development and deployment of precision-based therapeutics. In addition to the concerns for traditional antibiotics, such as toxicity, bioavailability, and feasibility of manufacturing, clinical use of precision antimicrobials will require rapid diagnostics to identify the patients for whom a particular therapy would be useful. Creation of an effective precision antimicrobial also demands a detailed understanding of the mechanisms that drive the infection cycle of a pathogen. This knowledge will inform the design of tailor-made drugs that prevent the virulence and/or persistence of a particular pathogenic organism by targeting pathways that are absent in the beneficial microbiota as well as the human host. Bacterial community dynamics must also be considered if precision antimicrobials are to be used in polymicrobial infections. These questions will require further investigation as the field of precision antimicrobials progresses.

Collaboration between academic laboratories and pharmaceutical companies will be instrumental in overcoming the unique challenges of precision antimicrobial development. Such collaborations already show promise in delivering precision antimicrobial therapies to the bedside. For example, Avidocins and mannosides are currently under development with the companies AvidBiotics (South San Francisco, CA) and Fimbrion Therapeutics (St. Louis, MO), respectively. Fimbrion Therapeutics is collaborating with GlaxoSmithKline (Brentford, London) to develop mannosides as an antibiotic-sparing therapeutic. A number of other anti-virulence therapies for *Staphylococcus aureus* and *Pseudomonas aeruginosa* are also undergoing clinical trials [10]. As the future of infectious disease therapeutics shifts to precision antimicrobials, it is imperative that large pharmaceutical companies increasingly engage in their research and development.

## Conclusions

The rise of antibiotic resistance, combined with a decades-long lull in the discovery of new antibiotics, indicates that we may run out of antibiotics to treat drug-resistant infections. Furthermore, we are only beginning to appreciate the inextricable links between the human microbiota and host health, and how antibiotic treatment alters this dynamic. Therefore, it is becoming increasingly evident that new therapeutic paradigms, including the use of precision antimicrobial therapies, must be employed to preserve human health. Precision antimicrobials offer a path to preserving therapeutic efficacy through the specific removal of targeted pathogens. The absence of off-target effects will reduce selective pressure on commensal microbes, while also preventing the disruption of key functions performed by the microbiota. To prevent pathogen resistance to antimicrobials, next-generation antimicrobials should be designed to kill or disarm microorganisms by targeting factors that are crucial for virulence. Successful use of these strategies for *E. coli*, *C. difficile*, *B. anthracis*, and others demonstrates a bright future for medicine as we enter a new era of targeted antimicrobial development.

## Abbreviations

UPEC: Uropathogenic *Escherichia coli*
UTI: Urinary tract infection
